# Development and Performance Characteristics of Personal Gamma Spectrometer for Radiation Monitoring Applications

**DOI:** 10.3390/s16060919

**Published:** 2016-06-21

**Authors:** Hye Min Park, Koan Sik Joo

**Affiliations:** Department of Physics, University of Myongji, Yongin 449-728, Korea; park2011@mju.ac.kr

**Keywords:** Ce-doped Gd–Al–Ga–garnet (Ce:GAGG), Si photomultiplier (SiPM), spectrometer, energy resolution, homeland security (HLS), environmental radiation monitoring system (ERMS)

## Abstract

In this study, a personal gamma (γ) spectrometer was developed for use in applications in various fields, such as homeland security and environmental radiation monitoring systems. The prototype consisted of a 3 × 3 × 20 mm^3^ Ce-doped Gd–Al–Ga–garnet (Ce:GAGG) crystal that was coupled to a Si photomultiplier (SiPM) to measure γ radiation. The γ spectrometer could be accessed remotely via a mobile device. At room temperature, the implemented Ce:GAGG-SiPM spectrometer achieved energy resolutions of 13.5%, 6.9%, 5.8%, and 2.3% for ^133^Ba at 0.356 MeV, ^22^Na at 0.511 MeV, ^137^Cs at 0.662 MeV, and ^60^Co at 1.33 MeV, respectively. It consumed only about 2.7 W of power, had a mass of just 340 g (including the battery), and measured only 5.0 × 7.0 cm^2^.

## 1. Introduction

Gamma (γ)-ray spectrometry analysis methods can be classified based on whether they involve direct or indirect detection, according to the manner in which the γ-rays are converted into electrical signals. The direct detection methods employ semiconductor devices that generate electrical signals immediately, with no intermediate stages, through γ-ray absorption. The indirect detection methods involve scintillation detection systems; in such a system, a photoelectric device detects electrical signals generated by photons with wavelengths in the visible range that are produced through interactions between the γ-rays and the scintillator.

CdZnTe, CdTe, and HgI_2_ are primarily used in room temperature semiconductor devices employing direct detection methods [1]. These materials are preferred in γ-ray spectrometry analysis because they yield energy resolutions superior to those achievable with scintillator crystals. However, the maintenance of these materials is more expensive than that of scintillator crystals, and their detection efficiencies and physical rigidities are very low [2]. Currently, compound semiconductor materials are also more expensive than scintillator crystals.

NaI(Tl), CsI(Tl), and LYSO(Ce) are the primary scintillator crystals used in indirect detection. These materials yield energy resolutions lower than those of semiconductors, but they are more sensitive. Because of the specific characteristics and limitations of each of these types of materials and the corresponding analysis methods, further development of detectors that combine scintillators and semiconductors is necessary and ongoing [3]. Thus, scintillator detectors remain promising candidates for use in homeland security (HLS) and environmental radiation monitoring systems (ERMSs).

In this study, a personal γ spectrometer was developed, and its feasibility was evaluated by analyzing its energy resolution using standard γ-ray sources. The device described herein, called the “Personal-Spect”, essentially consists of a scintillation detector, signal processing electronics, a voltage supply, signal analysis components, and display units.

## 2. Materials and Methods

An S12572-100C (photo-sensitive area: 3 × 3 mm^2^, Hamamatsu Photonics Co., Hamamatsu, Japan) Hamamatsu single Si photomultiplier (SiPM) was used in the Personal-Spect to minimize the overall system dimensions (Table 1).

**Figure 1:**
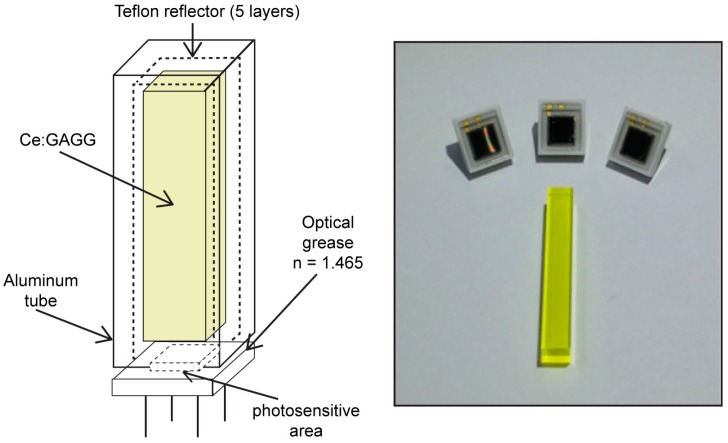
Structure of Ce-doped Gd–Al–Ga–garnet (Ce:GAGG) detector head (**left**); 3 × 3 mm^2^ Hamamatsu S12572-100C SiPM and 3 × 3 × 20 mm^3^ Ce:GAGG crystal (**right**).

The Personal-Spect prototype contained a 3 × 3 × 20 mm^3^ Ce-doped Gd–Al–Ga–garnet (Ce:GAGG) crystal, which was fabricated via the Czochralski crystal growth method using a seed crystal (Furukawa Denshi Co., Tokyo, Japan) and contained approximately 1% Ce. Ce:GAGG has a peak wavelength of 520–530 nm, which fits the spectral response range of the SiPM. Also, since this material has a density of 6.63 g·cm^−3^, its stopping power is high. Furthermore, its decay time (90 ns) is shorter than those of the conventional scintillators (CsI:Tl and NaI:Tl) [4]. In order to maximize the light output from the Ce:GAGG and to match it to the SiPM photosensitive area, the crystal geometry was optimized using Monte Carlo n-particle extended code [5]. Figure 1 shows the detector head of the Personal-Spect. The SiPM was coupled to a Ce:GAGG scintillator covered with five layers of a white diffusive (Teflon) reflector in order to optimize the scintillation light collection. The optical coupling was achieved by using optical grease (n = 1.465) as the coupling medium. The coupled detector was covered and sealed inside an Al tube to prevent background noise from external light.

**Figure 2:**
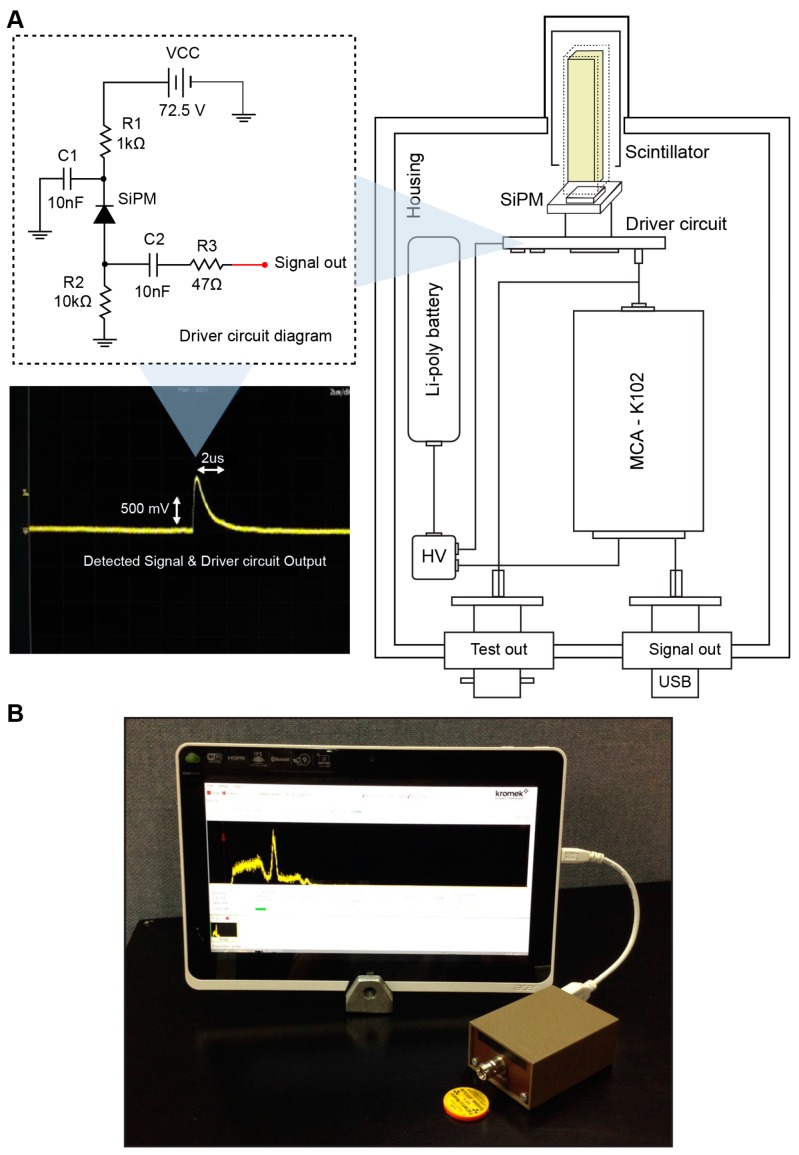
(**A**) Personal-Spect structure and (**B**) overall spectroscopy system.

Figure 2 shows the Personal-Spect structure and the overall spectroscopy system designed in this study. The fabricated Ce:GAGG-SiPM detector and electronics were installed in an Al shielding box to prevent background noise from external light.

Previously, charge-sensitive preamplifiers that convert scintillation detector charge signals into voltage signals, as well as shaping amplifiers that amplify converted voltage signals and simultaneously shape them into Gaussian forms, were designed for use in signal processing in γ-ray spectrometry [2]. However, such signal processing procedures cause signal attenuation due to impedance mismatch, decrease the signal-to-noise ratio through the amplification and shaping processes, and cause output signal loss due to detected signal overlap with the dead time.

Therefore, in this study, the signal processing stage was excluded due to the high electron amplification gains of SiPMs, and only the driver circuit of the scintillation detector was designed and built to analyze the output signal of the circuit. The left-hand side of Figure 2 shows a 700 mV pulse signal with a 2 μs pulse width that was generated by a ^22^Na source and measured using the driver circuit.

The voltage supply consisted of a main power supply and a voltage-boosting unit. The voltage-boosting unit was designed to increase an input voltage of 7.2 V up to 73 V ± 1.5 V, which is the input voltage of the SiPM. A Li-poly battery (7.4 V, 850 mAh) was used as the main power supply with a DC–DC converter module (UltraVolt, Inc., Ronkonkoma, NY, USA). This Personal-Spect had dimensions of 5.0 × 7.0 cm^2^, and it could run for more than 3 h with an average power consumption of 2.7 W.

The signal analysis was performed using a tablet computer, which constituted the display unit, and a miniature 4906-channel K102 Multichannel Analyser (Kromek Ltd., Sedgefield, UK).

In the experiments described herein, a standard disc-type radiation source was used to evaluate the Personal-Spect. Several standard γ-ray sources were used: ^133^Ba: 0.356 MeV, ^22^Na: 0.511 MeV and 1.27 MeV, ^137^Cs: 0.662 MeV, and ^60^Co: 1.17 MeV and 1.33 MeV (Spectrum Techniques, Oak Ridge, TN, USA). Each source had an activity of 1 μCi and was placed 10 mm from the surface of the developed device, and the live time was set to 300 s for each of the experiments.

## 3. Results and Discussion

**Figure 3:**
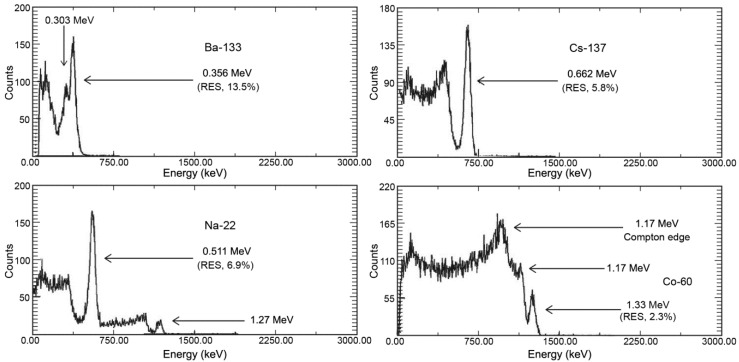
Measured energy spectra for ^133^Ba (0.356 MeV), ^22^Na (0.511 MeV), ^137^Cs (0.662 MeV), and ^60^Co (1.33 MeV).

Figure 3 shows the energy spectra obtained from the Personal-Spect. The 0.356 MeV peak of the ^133^Ba source exhibits an energy resolution of 13.5%. For the 0.511 MeV peak in the ^22^Na spectrum that was generated through positron annihilation during β decay, an energy resolution of 6.9% was measured. The 0.662 MeV peak in the ^137^Cs spectrum yielded an energy resolution of 5.8%, while for ^60^Co, an energy resolution of 2.3% was obtained at an energy of 1.33 MeV, and peaks at 1.17 MeV and 1.33 MeV are clearly observable [6].

**Figure 4:**
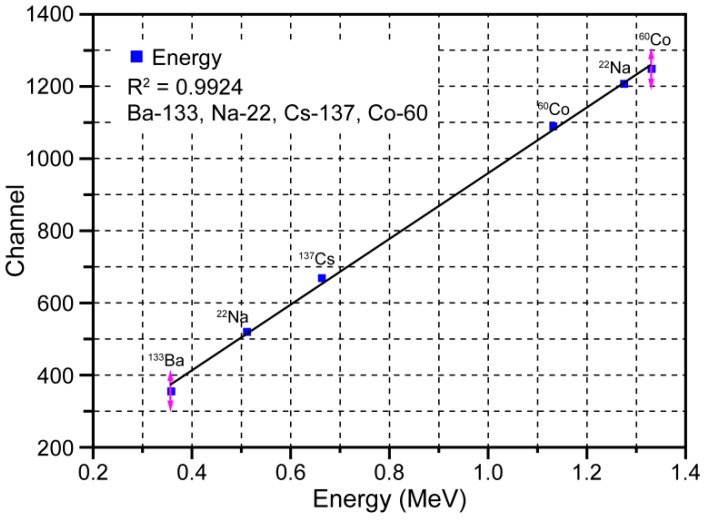
Linear fit of measured ^133^Ba, ^22^Na, ^137^Cs, and ^60^Co photopeak energies and channels.

Figure 4 depicts the Personal-Spect energy calibration line, which was used to evaluate the linearity of the γ-ray energy. The points on this graph were obtained and the line was estimated based on the measured ^133^Ba, ^22^Na, ^137^Cs, and ^60^Co photopeak energies and channels.

The fit shown in Figure 4 confirms the linearity of the relationship between the γ-ray energy and peak channel number for the energy range from 0.356 MeV to 1.33 MeV. The R^2^ value of the linear relationship was found to be 0.9924.

**Figure 5:**
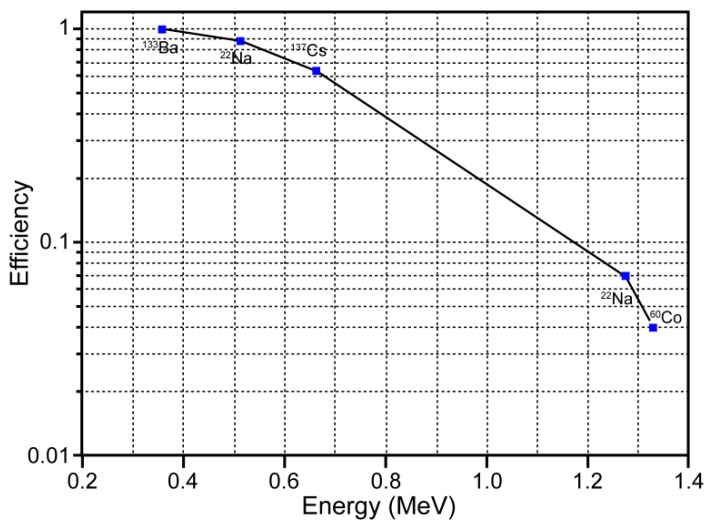
Full-energy peak efficiency in the 0.356–1.3 MeV energy range.

Figure 5 depicts the Personal-Spect detection efficiency curve, which was used to evaluate the full-energy peak efficiency (FEPE) for γ-rays of various energies. The FEPE is the efficiency with which γ-rays of a given energy produce full-energy peak pulses, rather than pulses of other sizes.

The efficiency curve in Figure 5 shows a relatively linear decrease within the high-energy range from 0.356 MeV to 1.33 MeV. Clearly, to be useful, the detector must be capable of absorbing a large fraction of the γ-ray energy. This capability can be realized by using a detector of suitable size or by choosing a scintillator material with a sufficiently high Z [7].

**Figure 6:**
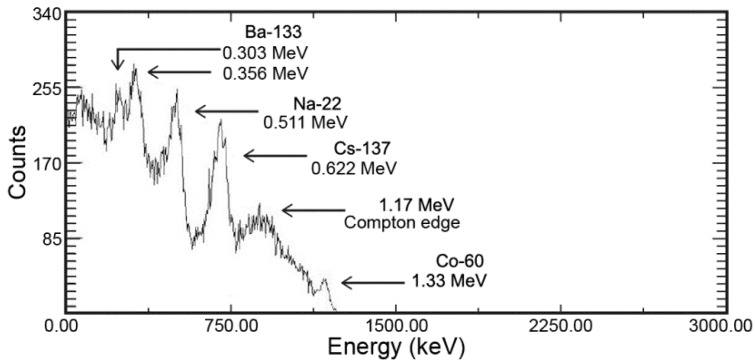
Energy spectra emitted from ^133^Ba (0.303 MeV and 356 MeV), ^22^Na (0.511 MeV), ^137^Cs (0.662 MeV), and ^60^Co (1.33 MeV).

Figure 6 depicts the γ-ray energy spectrum for a mixed source containing ^133^Ba, ^22^Na, ^137^Cs, and ^60^Co [8]. The results presented in this figure confirm that γ-ray spectrometry analysis is possible for a mixed source based on the γ-ray energy peaks in the range from 0.3 MeV to 1.3 MeV.

## 4. Conclusions

A personal γ spectrometer was fabricated and evaluated to assess its usability in radiation monitoring applications. This device is low-power, requiring only 2.7 W from a single 7.4 V input, has a mass of 340 g (including the battery), and has dimensions of 5.0 × 7.0 cm^2^. It can be accessed remotely via a mobile device. This device exhibited higher energy resolutions, as confirmed by the spectra obtained for several standard γ sources. The features of the Personal-Spect were characterized, and the best full-width at half-maximum energy resolution was found to be 5.8% at 662 keV. Thus, the Personal-Spect is expected to be applicable in fields such as HLS and ERMS as a personal γ spectrometer and to replace expensive semiconductor detectors.

## Figures and Tables

**Table 1:** Specifications of Si photomultiplier (SiPM) used in this study.

Parameter	Value
Photosensitive area	3 × 3 mm^2^
Number of pixels	900
Spectral response range	320–900 nm
Peak photon detection efficiency (at 450 nm)	35%
Bias voltage	Vbr + 1.4 V
Breakdown voltage	65 V ± 10 V
Gain	2.8 × 10^6^
Operating temperature	−20 °C–40 °C
